# Mutations in *nalc* gene of Mex AB-OprM efflux pump in carbapenem resistant *Pseudomonas aeruginosa* isolated from burn wounds in Yazd, Iran

**Published:** 2020-02

**Authors:** Fatemeh Akhavan Tafti, Gilda Eslami, Hengameh Zandi, Kazem Barzegar

**Affiliations:** 1 Department of Microbiology, School of Medicine, Shahid Sadoughi University of Medical Sciences, Yazd, Iran; 2 Institute for Advanced Research and Education in Transfusion Medicine, Yazd, Iran; 3 Department of Parasitology and Mycology, School of Medicine, Shahid Sadoughi University of Medical Sciences, Yazd, Iran; 4 Research Center for Food Hygiene and Safety, Shahid Sadoughi University of Medical Sciences, Yazd, Iran; 5 Department of Microbiology, School of Medicine, Shahid Sadoughi University of Medical Sciences, Yazd, Iran; 6 Department of English Language, School of Medicine, Shahid Sadoughi University of Medical Sciences and Health Services Yazd, Iran

**Keywords:** *Pseudomonas aeruginosa*, Carbapenem resistant, Efflux pump, *nalc* gene

## Abstract

**Background and Objectives:**

Burn wound infections have emerged as an important cause of morbidity and mortality in patients due to prolonged hospital stay. *Pseudomonas aeruginosa*, is the second cause of bacterial burn wound infections. Resistance mechanisms among *P. aeruginosa* are intrinsic or acquired. Intrinsic resistance mechanisms among *P. aeruginosa* isolates are inducible AmpC cephalosporinase, decrease of specific porin OprD, and overexpression of RND efflux pump. The aim of this study was detection of mutations in *nalC* gene in carbapenem resistant *P. aeruginosa* isolated from burn wounds.

**Materials and Methods:**

In this cross-sectional study, 180 burn-wound specimens were collected. Suspected lactose-negative colonies were identified by conventional biochemical methods. Kirby-Bauer and Etest methods were used for susceptibility testing. PCR and sequencing techniques were used for the detection of *nalC* mutation.

**Results:**

Out of 180 specimens received in the laboratory, 54 of isolates were isolated and identified as *P. aeroginosa* (30%). Of these isolates 20 (37%) were resistant to at least two carbapenems simultaneously. From these carbapenem resistant isolates, 19 (95%), 14 (70%), 14 (70%), 19 (95%) and 16 (80%) were resistant to imipenem, cefepime, piperacillin, ceftizoxime and gentamicin, respectively. Only 1 (2%) isolate was sensitive to all carbapenems and did not has mutation in *nalC* gene, 20 (37%) isolates were resistant to at least two carbapenems, and had mutations in *nalC* gene (Gly71▸Glu and Ser209▸Arg).

**Conclusion:**

As the results showed, mutation in efflux pump was observed in carbapenem resistant isolate and this confirmed that the indiscriminate use of antibiotics for treatment or prophylaxis can increase mutation in efflux pump.

## INTRODUCTION

Burn wound infections have emerged as an important cause of morbidity and mortality in patients due to prolonged hospital stay (1). Gram-positive bacteria are some of the first bacteria that colonize burn wounds, followed quickly by Gram-negative. *Pseudomonas aeruginosa* is the second cause of bacterial burn wound infections (2).

Resistance mechanisms among *P. aeruginosa* are intrinsic or acquired. Intrinsic resistance mechanisms among *P. aeruginosa* isolates are inducible AmpC cephalosporinase, decrease of specific porin OprD (3, 4) and overexpression of RND efflux pump. Over-expression of mexAB-OprM has caused the emergence of Multi-Drug Resistance (MDR) *P. aeroginosa* (5).

RND pumps typically exist as a tripartite system (6), consisting of a RND cytoplasmic membrane transporter, a Membrane Fusion Protein (MFP), and an Outer Membrane Protein (OMF) (7). This complex forms a channel, spanning the entire membrane, allowing for the proton-derived transport of lipophilic and amphiphilic drugs from the cytoplasm of the cell across the cytoplasmic membrane, peptidoglycan, and outer membrane through the periplasmic space (8). The MexAB–OprM system has the broadest substrate range among all of 10 characterized *P. aeruginosa* efflux pumps (9). Substrates of this pump include ß-lactams, ß-lactamase inhibitors, quinolones, macrolides, tetracyclines, chloramphenicol, novobiocin, sulfonamides, and trimethoprim (9, 10). Several regulatory loci influence the expression of the MexAB-oprM operon (11). The mexR gene is located directly up the stream of mexA and transcribed divergently from MexAB-oprM and encodes a repressor belonging to the MarR family of regulatory proteins. Also, *nalC* gene (also known as PA3721) (12) encodes a putative repressor of the TetR/AcrR family, whose genes are located up stream of operon-encoded PA3720-PA3719 genes, that is negatively regulated by NalC (13). Loss of NalC resulted in over-expression of PA3720-PA3719, and subsequent experiments demonstrated that PA3719 upregulates MexAB-oprM by interacting with MexR (12, 13).

The purpose of this study was the detection of mutations in *nalC* gene (second regulatory gene of mex AB-OprM system) in carbapenem resistant *P. aeruginosa* isolated from burn wounds.

## MATERIALS AND METHODS

**Bacterial strain and growth conditions.** In this cross-sectional study, 180 burn wound specimens were collected from burnt patients in a burn-hospital in Yazd, Iran. These patients stayed over a week in hospital and did not have a burn wound infection at the admission time. Specimens were immediately transferred to microbiology laboratory at School of Medicine, Shahid Sadoughi University of Medical Sciences, inoculated on MacConkey agar media (Merck-Darmstadt, Germany) and Cetrimide agars (Merck-armstadt, Germany), and were incubated for 16–18 h at 35 °C. Suspected lactose-negative colonies were identified by conventional biochemical methods. Sugar utilization in Oxidation-Fermentation (OF) medium (Merck-Darmstadt, Germany), production of oxidase enzyme, growth at 42 °C and pigment production tests were performed for the identification of *P. aeruginosa*.

**Antibacterial agent and susceptibility testing.** Susceptibility of *P. aeruginosa* isolates to Ertapenem (10 μg), Meropenem (10 μg), Imipenem (10 μg), cefepime (10 μg), piperacillin (10 μg), ceftizoxime (10 μg) and gentamicin (10 μg) (Mast, England) were performed by the disk diffusion method (Kirby-Bauer) on Mueller-Hinton agar (Merck-Darmstadt, Germany) according to CLSI protocols (14).

Meropenem Etest strips (biomerieux, France) were used for the performing of the MIC. Briefly, bacterial suspension equivalent to 0.5 McFarland turbidity tube was prepared and inoculated on Mueller-Hinton agar. Etest strips were placed on the medium. After incubation for 16–18 h at 35 °C, Eclipse intersection with strip was considered as the inhibitory concentration. *P. aeruginosa* ATCC 27853 was used for control of all phenotypic tests.

**Bacterial genomic DNA extraction.** The salting out method was used for bacterial genomic DNA extraction (15). Briefly, after overnight culturing of bacteria in the Trypticase Soy Broth (TSB; Merck-Darmstadt, Germany), they were washed with phosphate buffer saline in triple. Cells were lysed by NET buffer (NaCl, 50 mM; EDTA, pH 8, 10 mM, Tris-base, pH 7.6, 50 mM) and Sodium dodecyl sulfate (SDS) with final concentration of 1%. Purification and precipitation were performed using saturated salt and cold ethanol, respectively. The quality and quantity of extracted DNA were assessed by agarose gel electrophoresis and spectrophotometer, respectively. The samples were stored at −20 °C for the next steps.

**Detection of** ***nalC*** **gene by polymerase chain reaction.** PCR technique was performed for detection of *nalC* gene using specific primers (*nalC*-L> CCTGGACATGGTGATAGAACG-*nalC*-R> CGGGTCCTGAACGAACTCT) (https://www.ncbi.nlm.nih.gov/tools/primer-blast/).

The PCR reaction mixture contained 1× master mix (Amlicon, Denmark) (including 10 mM tris-base, 0.2 mM dNTP, 1.5 mM MgCl_2_, and 1U Taq DNA polymerase), 10 pmol of each primer, and 100 ng template DNA in a total volume of 20 μl. Amplification was performed using thermocycler (Quant biotech, England) with initial denaturation at 94 °C for 5 min, followed by 35 cycles denaturation step at 94 °C for 85 s, annealing at 52 °C for 85 s, and extension at 72 °C for 85s. The final extension was at 72 °C for 5 min. PCR products were analyzed using gel agarose. Electrophoresis alongside with 50 bp DNA ladder. The samples with single amplicon with 724 bp in length were sequenced and analyzed with BLAST and the bioinformatic software necessary for mutation analysis in Sequence Retrieved System (SRS) at ABI (https://www.ncbi.nlm.nih.gov/tools/cobalt/re_cobalt.cgi) (16).

## RESULTS

Out of 180 burn wound specimens, 54 (30%) isolates were identified as *P. aeroginosa*. These were isolated from 36 men (67.6%) and 18 women (33.3%). Twenty (37%) isolates were resistant to at least two carbapenems (ertapenem, meropenem and imipenem) simultaneously. Among carbapenem resistant isolates, over 70% of them were resistant to ertapenem, meropenem, imipenem, cefepime, piperacillin, ceftizoxime and gentamicin (Table 1).

**Table 1.** Antimicrobial susceptibility pattern of carbapenem resistant *P. aeruginosa* isolates.

**Antibiotic**	**Piperacilin N (%)**	**Ertapenem N (%)**	**Imipenem N (%)**	**Gentamicin N (%)**	**Cefepim N (%)**	**Meropenem N (%)**	**Ceftizoxime N (%)**
Sensitive	6 (30)	2 (10)	1 (5)	3 (15)	5 (25)	1 (5)	1 (5)
Intermediate	0 (0)	0 (0)	0 (0)	1 (5)	1 (5)	1 (5)	0 (0)
Resistant	14 (70)	18 (90)	19 (95)	16 (80)	14 (70)	18 (90)	19 (95)

**Fig. 1.**
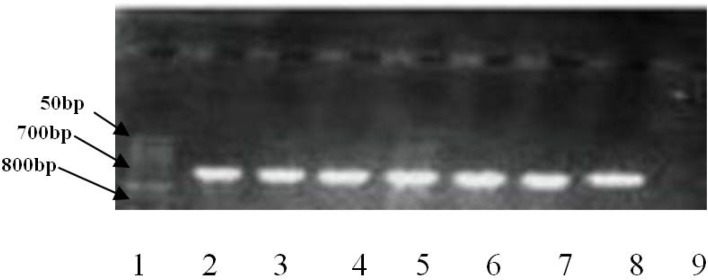
Agarose gel electrophoresis image for amplification analysis of *nalC* gene. Lane 1: 50 bp DNA ladder; lanes 2–8: Isolates with *nalC* (724 bp) and lane 9: negative control.

Isolates with MIC>16 μg/ml, according to the manufacturer's instructions, were considered as resistant to meropenem. In this way, out of 54 *P. aeroginosa* isolates, 35 (64.8%) were resistant to meropenem. Four isolates that were resistant to meropenem by disk diffusion method, had MIC<2 μg/ml, and were, therefore, considered sensitive. Only one sample was sensitive to all carbapenems and did not have mutation in *nalC*. Twenty (37%) isolates were resistant to at least two carbapenems and had mutation in *nalC.* Gel electrophoresis of *nalC* gene is shown in Fig. 1. *nalC* gene mutations (Gly71▸Glu and Ser209▸Arg) were observed in 22 resistant isolates. *nalC* mutation was not seen in susceptible isolates.

Among the isolates that had mutation in *nalC* gene, the strains 2ss, 9ss, 23ss were registered at GeneBank under accession numbers KP774795, KT624617 and KT624618, respectively.

## DISCUSSION

Burn wound infections as nosocomial infections are an important cause of mortality and disability after burns. *P. aeruginosa* is an opportunistic pathogen that grows well in moist environments at hospitals. Low sensitivity to antibiotics is one of the characteristics of these bacteria that make them difficult to treat. Carbapenems are used for treatment of infections caused by *P. aeruginosa* and over-expression of mexAB-OprM has led to the emergence of MDR *P. aeroginosa* (17). In this study, out of 20 carbapenem-resistant isolates, 18 (90%), 18 (90%), 19 (95%), 14 (70%), 14 (70%), 19 (95%) and 16 (80%) isolates were resistant to ertapenem, meropenem, imipenem, cefepime, piperacillin, ceftizoxime, and gentamicin, respectively. Ahadi et al. (2012) reported that out of 100 clinical isolates, 56, 59, 61, 65, 55, 57, 60, 62, 100 and 48% of them were resistant to ciprofloxacin, gentamicin, tobramycin, amikacin, imipenem, cefepime, ceftazidime, ceftriaxone, cefotaxime, oxacillin and piperacillin, respectively (18). Mir Salehian et al. (2011) reported that among 170 *P. aeruginosa* strains isolated from burn wounds in Tehran, Iran, the most resistant were observed against aminoglycosides and 52.9% of isolates were resistant to imipenem (2). In another study conducted in Esfahan, out of 98 *P. aeruginosa* isolates, the most resistance were observed against cefepime (91%), cefotaxime (95%) and ceftizoxime (85.7%) (1). Gill et al. (2011) in Pakistan reported that 90.3% and 85.4% of *P. aeruginosa* isolates were resistant to imipenem and meropenem, respectively, whereas in this study, 95% and 70% of isolates were resistant to imipenem and meropenem, respectively (20). Considering that in the Burns Hospital of Yazd, imipenem is one of the most frequently prescribed antibiotics for the treatment of hospitalized patients and since imipenem is prescribed as prophylaxis for the prevention of burn wounds infection, therefore, increasing resistance to this antibiotic is inevitable. Yet, it could be indicative of increased resistance to this class of antibiotics because of indiscriminate usage.

In present study, 35 (64%) of isolates had MIC>16 μg/ml for meropenem, whereas in China, more than 70% of *P. aeruginosa* isolates were susceptible to imipenem and meropenem (19). Gill et al. (2011) showed that 90.3% and 85.4% of *P. aeruginosa* strains in Pakistan were resistant to imipenem and meropenem, respectively (20). In a study in New York, USA, resistance rate of *P. aeruginosa* isolates against meropenem was 28% (3). It seems that the resistance pattern to antibiotics based on the method and amount of antibiotic usage is different in the various countries.

In this study, 20 (37%) isolates were resistant to at least two carbapenems and had mutation in *nalC.* Also, *nalC* mutations (Gly71▸Glu and Ser209▸Arg) were observed in isolates. In the study by Sadeghifard et al. (2012) in Tehran, Iran, 87.1% of resistant *P. aeruginosa* isolates had *nalC* mutation (21). Quale et al. (2006) reported that 33 carbapenem-resistant *P. aeruginosa* isolates were detected for the presence of *nalC* mutations in New York, USA. Gly71 ▸Glu, Ala145▸Val and, Ser209▸Arg mutations were observed in their study (3). In our study, substitution mutations (Gly71▸Glu and Ser209▸Arg) were observed in resistant isolates and, *nalC* mutations were not seen in sensitive isolates. Our substitution mutations that were seen in our study were similar to Quale`s results.

## CONCLUSION

The results of this study shows resistance to carbapenem and cephalosporins that are used for the treatment of patients with infections caused by *P. aeruginosa* are growing in our country. In present study, more than 70% of *P. aeruginosa* isolates were resistant to ceftizoxime, imipenem, gentamicin, and piperacillin. Detection of these resistant organisms, implementation of strict antimicrobial policies, and infection control programs such as antibiotic stew-ardship may prevent the rapid dissemination of these organisms.

The mutation in efflux pumps causes multi-drug resistant strains. Mutations in efflux pump were observed in resistant isolates and this confirmed that the indiscriminate use of antibiotics for treatment or prophylaxis can increase mutation in efflux pumps. In order to obtain more complete results in this area, it is necessary to conduct more studies about changes in gene expression and its relationship with the *nalC* mutations.
